# Fostering Circular Economy: Brewing By-Products as Innovative Ingredients for Cereal Bar Formulation

**DOI:** 10.3390/foods13152355

**Published:** 2024-07-26

**Authors:** Maria Paciulli, Giovanni Sogari, Margherita Rodolfi, Ottavia Parenti, Giulia Andreani, Emma Chiavaro

**Affiliations:** Department of Food and Drug, University of Parma, 43124 Parma, Italy; giovanni.sogari@unipr.it (G.S.); margherita.rodolfi@unipr.it (M.R.); ottavia.parenti@unipr.it (O.P.); giulia.andreani@unipr.it (G.A.); emma.chiavaro@unipr.it (E.C.)

**Keywords:** snack bars, upcycled ingredients, storage, sensory analysis, consumer perception

## Abstract

Brewer’s spent grain (BSG) was used as a sustainable and healthy ingredient in two cereal bar formulations, with honey (H) and chocolate (C) used as the binding systems’ characterizing ingredients. The two bars, formulated using three levels of BSG (H1: 8.5%; H2: 12.7%; H3: 21.2%; C1: 3.9%; C2: 7.7%; C3: 15.5%) and stored for 20 days, were studied from a physicochemical perspective and compared to non-enriched control bars. The analysis showed that BSG enriched the bars with minerals, B vitamins, proteins, and fibers, meeting the required contents for the “high fiber” nutritional claim. Moisture content and water activity decreased with increasing BSG quantity and storage time. Higher BSG content increased flexibility in H bars after 7 days, while decreasing water content and increasing hardness in C bars at 1 storage day. Higher BSG levels darkened the samples’ color with little change during storage. In addition, a consumer sensory test was conducted. The results showed that providing information on BSG had little impact on liking, purchase intent, and sensory perception. In addition, under blind conditions, H bars were considered more natural and healthier than the C bars; however, these differences were not significant in the informed conditions. This study shows the potential use of upcycled ingredients in cereal bars and highlights the central role of the sensory experience on consumer appreciation, considering also information provision.

## 1. Introduction

Beer is the third most consumed beverage in the world and, in many countries, it is considered a traditional product linked to the local gastronomic culture [1].

Nevertheless, the beer market is continuously changing, and, over the last 50 years [2], beer consumption has spread from traditional beer-drinking areas (e.g., UK, Belgium, and Germany) to other countries that were not traditionally linked to beer consumption, such as Spain and Italy [3]. In the latter two countries, beer consumption has risen, whereas wine consumption has decreased [3]. Specifically, Italy is currently among the countries with the highest number of breweries per capita worldwide [4].

At the same time, the brewery sector is also responsible for the production of a great amount of waste derived from spent malt [5]. In fact, during the brewing process, malted barley is milled and mixed with hot water. The water temperature enables the enzymatic hydrolysis of malt elements (starch, proteins, β-glucans, and arabinoxylans), which are converted into fermentable and non-fermentable sugars, polypeptides, and amino acids [6]. In this process, the derived liquid (wort), which represents the first step in the beer production process, is separated from the solid fraction, which is known as brewer’s spent grain (BSG) [7]. BSG contains husk (cellulose), non-cellulosic polysaccharides, lignin, some insoluble proteins (mainly hordeins and glutelins), and lipids [8,9].

From the brewery process, BSG is the most abundant residue, with an estimated amount of 37.2 million tons produced worldwide in 2021 [10]. Currently, this by-product is mostly used as animal feed or landfilling [7]. However, BSG possesses several positive characteristics; it is indeed a “clean” by-product and does not require purifications or extraction to be reintroduced in food production. Moreover, BSG has a valuable nutritional profile—e.g., high fiber, vitamin, and β-glucan contents [8]. It is well known that dietary fibers can reduce fat assimilation and the glycemic index [11]. Given BSG’s high fiber content, it can be considered a potential ingredient in the production of food products, especially bakery goods [12]. For example, Waters et al. (2012) studied the effect of fermented BSG on bread production. Technological results showed that the addition of fermented BSG significantly improved the textural properties of bread, and sensorial acceptance by panelists was obtained for a maximum of 10% BSG incorporation into the bread formulation [13]. Other authors tested BSG incorporation in cookie formulations [14]. For example, Guo, M. et al. (2014) found that the incorporation of 10% BSG led to higher bulk density, protein content, and sensory acceptance in comparison with the control [14]. In addition, Cuomo et al. (2022) focused on the development of high-fiber pasta with BSG incorporation [15]. The results from the technological and nutritional perspective were promising since BSG-enriched pasta showed positive characteristics in terms of color, texture, and cooking quality compared to 100% semolina and 100% wholegrain semolina pasta.

Cereal snacks are another food category that could be enriched with BSGs. The snacks market has experienced rapid growth due to evolving consumer lifestyles favoring convenient on-the-go products, such as cereal bars. These products are also ideal food carriers to be fortified with proteins, fibers, and other nutraceutical elements [16,17]. In recent years, the cereal snack market has shifted from traditional unhealthy options to healthier alternatives. Cereal bars have indeed emerged as one of the most popular choices, playing a pivotal role in meeting consumers’ increasing health and natural consciousness [18]. The incorporation of food by-products, which often contain significant amounts of valuable nutritional elements such as antioxidants, dietary fibers, and minerals, into cereal bar formulations represents a strategy to enhance the nutritional profile of snacks available in the market [19,20]. In this context, previous studies [21,22] showed that cereal bars would be an appropriate food category to be enriched with BSG.

The upcycled food market has grown in recent years [19]; nevertheless, consumers may still link the concept of “upcycling” with negative associations [21,23]. In particular, the low familiarity with and lack of awareness of BSG could be a barrier to preventing consumers from purchasing foods containing this upcycled ingredient [12,21]. Delivering the proper messages about sustainability and nutritional properties could be a valid strategy to increase consumer acceptance of BSG-enriched food products [22].

Another critical aspect to take into account when considering consumers’ acceptance and purchasing behavior is the sensory characteristics of the product. Although in the literature, some articles investigated the development of snacks enriched with BSG, only few studies included a sensory analysis of the final product [19]. Furthermore, while previous research provided valuable insights into the role of communication strategies for upcycled food [23], there is a gap in the existing literature regarding the effect of providing information on the nature of the upcycled ingredient. Curutchet et al. (2022) investigated the sensory profiles of bread, pasta, and chocolate milk enriched with BSG; however, the authors only considered the blind condition, without informing participants about the product characteristics (i.e., use of upcycled ingredients) [24]. Therefore, there is a need to understand how the sensory perception might change when consumers are informed about the addition of BSG to the product formulation.

The present study aimed to explore the possibility of reintroducing BSG in the food chain by developing two cereal bars, formulated with different binding systems, and performing a physicochemical characterization of the tested samples at three different storage times. In addition, the two BSG-enriched cereal bars were tested in a consumer sensory test using a between-subject design with a blind and an informed condition.

## 2. Materials and Methods

### 2.1. Materials

#### 2.1.1. Brewers Spent Grains (BSG)

The Italian brewery “Birrificio HIBU Srl” in Burago di Molgora, MB, Italy, provided the BSG from the production of a high-fermentation lager beer. Following the mashing process, the wet BSGs with a moisture content of approximately 80% were frozen until needed. Then, the BSGs were dried at 48 °C for 48 h [22] to prevent protein denaturation and the formation of undesirable flavors. After the drying process, a milling step was performed using a grinder mixer (Oster^®^ 12-Speed Blender, Osterizer, Milwaukee, WI, USA) in order to grind the BSGs to a final particle size of around 850 μm.

#### 2.1.2. Cereal Bars Formulation and Storage Conditions

Cereal bars were produced at the Food Technology Laboratory of the University of Parma (Italy). Solid ingredients for the two types of cereal bars, purchased at a local market (Parma, Italy), included puffed rice (Sarchio), puffed quinoa (Sarchio), oat flakes (Sarchio), raisins (Noberasco) [raisins (99.5%) cottonseed oil (0.5%)], and cranberries (Select Horeca) [cranberries (60%), sugar (39%), sunflower oil (1%)].

Two types of cereal bars were developed, which were characterized by two different binding systems, namely “H” (honey binding system) and “C” (chocolate binding system) (Table 1); different levels of BSG incorporation; and different amounts of solid ingredients (Table 2). The selection of the incorporation levels for both H and C bars was based on preliminary trials. At first, since the aim of this study was to promote the valorization of beer by-products, the maximum adequate level of BSG incorporation was determined. This level was selected based on the capability of the binding system to effectively hold all the ingredients together, considering the higher hygroscopicity of BSG in comparison to the other solid ingredients. Then, two other levels were included, the selection of which was influenced by the type and the amount of the other ingredients used in the two formulations.

The results of the preliminary trials allowed us to obtain the following BSG incorporation levels: 0.0%, 8.5%, 12.7%, 21.2% weight/total weight in H bars, and 0.0%, 3.9%, 7.7%, 15.5% weight/total weight in C bars. The cereal bars were coded as follows: HSTD = standard cereal bar formulated with binding system H and without the addition of BSG; H1 = cereal bar formulated with binding system H and 8.5% BSG; H2 = cereal bar formulated with binding system H and 12.7% BSG; H3 = cereal bar formulated with binding system H and 21.2% BSG; CSTD = standard cereal bar formulated with binding system C and without the addition of BSG; C1 = cereal bar formulated with binding system C and 3.9% BSG; C2 = cereal bar formulated with binding system C and 7.7% BSG; C3 = cereal bar formulated with binding system C and 15.5% BSG.

*H binding system*—For the preparation of the H binding system, soy lecithin was put in water for approximately 1 min; the obtained mixture was mixed on a heating plate (Schott CERAN, GWM) at temperature level 4. Once completely dissolved in water, the mixture of soy lecithin and water was added with all the other ingredients of the H binder (margarine, sugar cane, and honey). The ingredients were mixed on the heating plate at temperature level 7 for 2 min, reaching the boiling state of the whole mixture.

*C binding system*—For the preparation of the C binding system, soy lecithin was put in half of the water amount for approx. After 1 min, the obtained mixture was mixed on the heating plate at temperature level 4. Separately, margarine and white chocolate were melted and mixed at a temperature level 4 with the soy lecithin mixture, sugar cane, and sunflower oil. Then, cornstarch was added and mixed on a heating plate at temperature level 7 for 5 min. During cooking, the remaining water amount was added to the product.

In both H and C cereal bars, solid ingredients were added and mixed with the binding systems when the latter were still warm, without the use of the heating plate. The obtained mixtures of both H and C formulations were pressed in a mold (height: 5 cm; length: 9 cm; width: 1 cm) up to complete filling, then allowed to rest at 4 °C for 30 min. The final weights of the cereal bars were 24.50 g for the H bars, and 31.00 g for the C bars.

The obtained cereal bars were then stored in a thermostatic chamber (Memmert Schwabach, Germany) at T = 25 °C for 20 days, without any packaging. Physicochemical analyses were performed after 1 (t1), 7 (t7) and 20 (t20) days from the production process.

### 2.2. Methods

#### 2.2.1. Chemical Composition of Brewers’ Spent Grain (BSG)

The proximate composition of BSG was assessed by using official methods set up for vegetable matrices. Moisture was measured according to the official ISTISAN 96/34 [25] method by weighing the sample before and after drying it in the oven at 105 °C for 24 h and calculating the percentage of water lost. Total nitrogen was determined according to the official ISO1871:2009 [26] method with a Kjeldahl system; from the total nitrogen value, the protein percentage was calculated using 6.25 as a nitrogen-to-protein conversion factor. Crude fat content was determined according to the Reg. CE 152/09 H—protocol B method. Total dietary fibers were determined using the enzymatic–gravimetric official AOAC 985.29 [27] method. Digestible carbohydrates were determined by difference. Crude ash was determined according to the Reg. CE 152/09 M [28] method. Total sugar content was determined after inversion according to the Reg (CE) N. 152/2009 J [29] method.

The amino acid profile of BSG proteins was measured using HPLC according to the official AOAC 1990 method 982.30 [30]. Saturated, unsaturated, and trans fatty acids were determined according to the official UNI EN ISO 5509/1998 [31] and UNI EN ISO 5508/1998 [32] methods.

The concentrations of Na, NaCl, Ca, Mg, Fe, P, K, Mn, Zn, Al, Cu, Co, and S were determined according to the official EN ISO 6869/2001 [33] method. Thiamine (vitamin B1), riboflavin (vitamin B2), pantothenic acid (vitamin B5), pyridoxine (vitamin B6), Cobalamin (vitamin B12), Nicotinic acid, and Nicotinamide (vitamin PP) were measured according to Sim et al. [34].

#### 2.2.2. Physicochemical Analyses of the Binding Systems

The physicochemical analyses performed on the two different binding systems included the analysis of water activity (*a*_w_) and moisture content (MC).

Water activity was measured at 25 °C using Aqualab 4TE (Decagon Devices, Pullman, WA, USA). For the measurements, the binding systems were carefully pressed in the *a*_w_ cups; three replicates were performed for each binding system.

MC (%, g of water/100 g of sample) of both binding systems was measured following the refractometric method [35]. A digital refractometer (TDR 095C) was used to determine the total solid content using the refractive index (at T = 20 °C) minus unity; the MC corresponded to 100 minus the percentage of total solid content. Four measurements were performed for each binding system.

#### 2.2.3. Physicochemical Analyses of the Cereal Bars

Physicochemical analyses of cereal bars were performed at three different storage times (t1, t7, and t20). The following physicochemical analyses were performed: water activity (*a*_w_), moisture content (MC), and texture and color analysis.

Water activity was measured at 25 °C using Aqualab 4TE (Decagon Devices, Pullman, WA, USA). For the measurements, cereal bars were milled and carefully pressed in the *a*_w_ cups; three replicates were performed for each cereal bar.

MC (%, g of water/100 g of sample) of cereal bars was measured by gravimetry at 105 °C until a constant weight was obtained, according to the standard method AACC 44–15.02; three replicates were performed for each cereal bar.

The texture properties of cereal bars were measured with a texture analyzer (TA.XT2 Texture Analyzer) equipped with a 30 kg load cell (Stable Micro Systems, Godalming, UK). A cutting test was performed according to the methods described by Kim et al. (2009) [36] to measure the maximum cutting force (N), as a reference for fracturability, and distance at the maximum force (mm), as a reference for deformability. All cereal bars were cut in the central part of the sample. The parameters used for the analysis were as follows: pre-test and post-test speed = 5.00 mm/s, test speed = 2.00 mm/s, strain 70%, trigger force = 0.100 N. The analysis was performed on five replicates for each type of cereal bar (H and C bars).

The cereal bars’ color was measured by the software Image J-NIH (Image J software v. 1.38x, Media Cybernetics Inc., Rockville, MD, USA) on digital images acquired with a scanner (Hewlett Packard, Palo Alto, CA, USA) (resolution of 600 dots per inch). From the digital images, the average color value, given by the contribution of the individual components on the surface of the cereal bars, was obtained and expressed as red, green, and blue (RGB) values. The data were then switched to the Commission Internationale de l’Eclairage (CIELAB) system by obtaining the color parameters of lightness (L*), red/green index (a*), and blue/yellow index (b*). Nine replicates for each type of cereal bar (H and C bars) were performed.

### 2.3. Sensory and Consumer Central Location Test

All procedures involving human subjects underwent review and approval by the University of Parma Institutional Review Board. Participants received written consent forms and were given the chance to inquire with researchers before their involvement.

The cereal bars selected for sensory analysis included only the samples H2-t20 and C2-t20. The selection criteria were based on two factors: (i) level of BSG enrichment and (ii) storage time. For the level of BSG enrichment, an intermediate BSG level was chosen to achieve the “high fiber content” claim for both H and C bars. Additionally, intermediate BSG levels for both formulations were considered more acceptable by an internal group of 10 semi-trained panelists. They were individuals who underwent 1 h training, receiving guidance on the evaluation of texture, visual, and flavor parameters. For storage time, the longest storage time (t20) was selected as it represented the minimum interval time required for the cereal bars to reach the appropriate physicochemical stability.

#### 2.3.1. Central Location Test Methodology

The samples were tested in a 30 min session and presented to the panelists at room temperature on disposable plates. The two samples were identified with randomly assigned three-digit codes. The panelists were randomly divided into two groups: in the first group (blind group), the samples were presented as “cereal bars” and no other information was provided, whereas the second group (informed group) was given additional information about the product.

We provided specific information regarding upcycled food products instead of vague and abstract communication, i.e., we defined what BSG is and how this could be used in the food industry. In detail, the information provided to the informed group was the following: “The cereal bars you are going to taste have been made using by-products from the beer industry (brewers’ spent grain). These cereals are considered by-products and despite their high nutritional content, these grains are typically reused in non-food applications. In this case, we aimed at valorizing BSG as an ingredient in the formulation of sustainable bars”.

The order of sample evaluation was randomized and counterbalanced across the study population. Filtered water and unsalted crackers were available to panelists as palate cleansers.

#### 2.3.2. Study Population

The sensory experiment was conducted at the University of Parma with 76 young Italian consumers (18–35 years old). We focused on the young population as they might have been exposed to upcycled food more than older consumers [23]; moreover, the chosen food category (cereal bars) is commonly consumed in this age group [37]. Individuals with food allergies were excluded from participation and no incentive was offered to panelists. The blind group comprised 41 participants (female 56.1%; 22.5 ± 3.8 years old), whereas the informed group included 35 young consumers (female 31.4%; 23.7 ± 3.8 years old).

#### 2.3.3. Questionnaire Structure

Upon their arrival, panelists filled out a paper-based survey to gather general socio-demographic information. Successively, each participant was randomly assigned to either the blind or informed group.

In both groups, panelists rated their expected (before tasting) and actual (after tasting) liking for both H2-t20 and C2-t20 cereal bars by using a 7-point hedonic scale, from 1 = dislike extremely to 7 = like extremely [38]. After tasting each product, participants were asked to rate specific sensory characteristics (i.e., aspect, color, texture, crunchiness, chewiness, adhesiveness, taste, sweetness, and smell) on a 9-point hedonic scale [39]. In addition, check-all-that-apply (CATA) questions [40,41] were used to further explore the sensory descriptors of both H2-t20 and C2-t20 cereal bars. Finally, participants’ purchasing intent for the proposed products and for a general energy bar made with by-products of the beer or cereal industry was investigated using a scale from 1 = definitely would not buy to 5 = definitely would buy [42].

### 2.4. Data Processing

For physicochemical analyses, the statistical software SPSS Statistics (Version 27.0, SPSS Inc., Chicago, IL, USA) was used to calculate means and standard deviations. One-way ANOVA was performed using the same software to assess significant (*p* < 0.05) differences due to the independent variable’s formulation and storage. The Tukey HSD test was used as the post hoc test. Pearson correlation coefficients were calculated between the dependent variables at 95% and 99% confidence levels (*p* < 0.05 and *p* < 0.01), considering the effects of both BSG level and time of storage.

R statistical software version 4.3.2 [43] was used to perform statistical analyses related to the consumer central location test. To investigate differences in consumers’ expected and actual liking between products and conditions, and differences in their purchasing intention of the two cereal bars, two-way ANOVAs were performed. For all scaled hedonic and the remaining purchase intent questions, a series of *t*-tests were performed. Frequency counts and percentages were calculated for the CATA questions. Within each group (blind and informed), Cochran’s Q tests (with Sheskin as a post hoc test) were conducted to determine the presence of differences between the two products (*p* < 0.05).

## 3. Results and Discussion

### 3.1. BSG Chemical Composition

In Table 3, the proximate composition of BSG is reported in dry weight, considering a starting moisture content of 79.42%. BSG is indeed very wet when coming out from the factory; therefore, it needs a drying step to be stabilized and then used as an ingredient [44]. Fibers and proteins represented the most abundant components of BSG, as already reported by other authors [45].

The protein fraction also revealed a good amount of essential amino acids (Table 4). Among minerals (Table 3), Phosphorus, Calcium, Sulfur, and Magnesium were the most represented ones, with values of 5638, 4307, 2398, and 2086 mg/kg, respectively, which is in line with previous studies [8]. The vitamin quantification (Table 3) revealed a large amount of B vitamins, with B3 vitamin being the most abundant one (44.47 mg/kg). B3 vitamin, also known as PP vitamin or niacin, is composed of Nicotinic acid and Nicotinamide, and it is a water-soluble compound known as a pellagra-preventing factor [46]. Fărcaș et al. (2022) studied the in vitro bioaccessibility of B group vitamins from different BSGs and concluded that vitamins B12 and B1 have higher bioaccessibility compared with vitamins B3 and B6 [47].

Fiber represented approximately 60% of the total BSG composition, making this by-product a valuable ingredient for food formulations aimed to be claimed as a “source of fibers” or “with high fiber content”, according to European legislation [48]. In this study, the inclusion of BSG in the cereal bar formulations at certain levels allowed the cereal bars to meet the nutritional claim “with high fiber content”, as reported in Table 5.

### 3.2. Moisture Content and Water Activity

The moisture content (MC) and water activity (*a*_w_) of the cereal bars were mostly influenced by the composition of their binders, as shown in Table 6. The H binder had a simpler recipe compared to the C binder. Both binding systems showed similar MC levels, ranging from 70 to 72%, and exhibited notably different *a*_w_ levels (H binder: *a*_w_ = 0.35; C binder: *a*_w_ = 0.68) (Table 6).

MC and *a*_w_ of H and C binders largely impacted the values of these parameters when measured in the corresponding cereal bars (Table 6). MC of the H bars at t1 had values of around 9%, with no significant differences when comparing the HSTD bar with the BSG-enriched bars. Conversely, the *a*_w_ value of the H bars at t1 was significantly influenced by the formulation and showed an inverse trend with the amount of BSGs in the recipe. This trend ranged from 0.45 ± 0.03 of the HSTD to 0.37 ± 0.01 of H-3 (~18% drop). The values of MC and *a*_w_ found in these samples were in line with those reported by Su-Ah et al. (2018) [49] on low-calorie cereal bars formulated with different saccharin levels. The higher hygroscopicity of BSG in comparison to the other ingredients of cereal bars may justify the lower *a*_w_ of the BSG-enriched bars [50]. In C bars at t1, the values of both MC and *a*_w_ decreased by around 39% and 38%, respectively, when the amount of BSG in the cereal bar formulation was increased (Table 6). The higher *a*_w_ observed in the C bars when compared to the H bars may be attributed to the higher sugar content in the H binder (also coming from honey) which has a high water-binding capacity, and to the higher fat content in the C binder, which has a lower water retention capacity. Fats indeed interact with the BSG protein during mixing, enhancing fat absorption and consequently resulting in higher water availability in the sample [51]. Moreover, the lower BSG amount and total solid ingredients, as well as the higher binder/solid ingredients ratio of the C bars compared to the H bars (Table 1 and Table 2), may have also contributed to this result.

During the 20 days of storage at T = 25 °C, a gradual decrease in MC was observed in all cereal bars (Table 6). Significant differences were noted in the H1 bars and all C bar samples, particularly noticeable from t1 to t7, which were attributed to water evaporation. Concerning *a*_w_, a gradual and significant reduction was noted during storage for the STD samples, with approximately 45% and 60% drops for the H and C-STD bars, respectively, from t1 to t20. In the BSG-enriched samples, different behaviors were observed according to the formulation. H1, H2, and H3 initially showed a significant decrease in *a*_w_ after 7 days, followed by a subsequent increase between t7 and t20. This pattern, already observed in biscuits during storage [52], may be linked to initial water evaporation, followed by further water redistribution among the components.

Among the C bars containing BSG, only C3 showed a behavior similar to the H bars, also having *a*_w_ levels comparable to the H formulations. For C1 and C2, starting from higher *a*_w_ levels, the phenomenon of water evaporation was dominant in comparison to water redistribution; indeed, *a*_w_ tended to decrease during the storage time.

### 3.3. Texture

**Figure 1:**
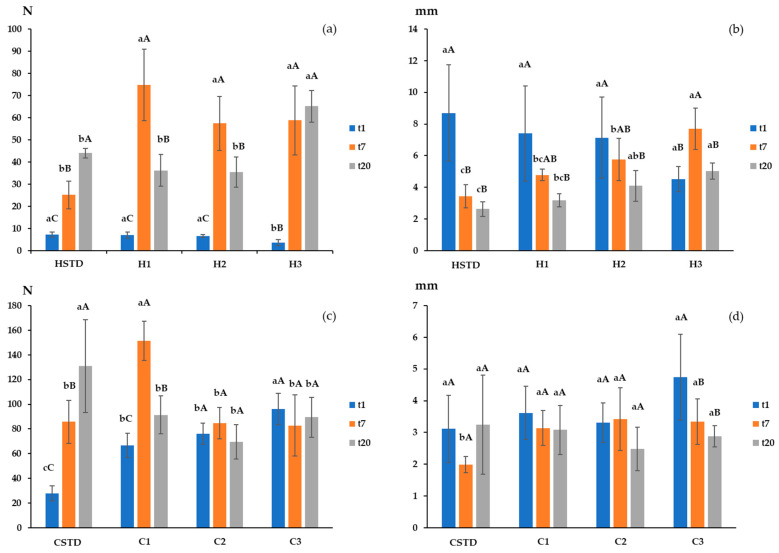
Fracturability (**a**,**c**) and deformability (**b**,**d**) of cereal bars. Note: Data expressed as mean values ± standard deviations. (**a**) Fracturability (N) for H bars; (**b**) deformability (mm) for H bars; (**c**) fracturability (N) for C bars; (d) deformability (mm) for C bars. Different small letters in the same bar indicate significant differences (*p* < 0.05) among formulations at the same storage time. Different capital letters in the same bar indicate significant differences (*p* < 0.05) for the same formulation at different storage times. HSTD = standard cereal bar with H binding system and without BSG; H1 = cereal bar with H binding system and 8.46% BSG; H2 = cereal bar with H binding system and 12.69% BSG; H3 = cereal bar with H binding system and 21.15% BSG; CSTD = standard cereal bar with C binding system and without BSG; C1 = cereal bar with C binding system and 3.87% BSG; C2 = cereal bar with C binding system and 7.74% BSG; C3 = cereal bar with C binding system and 15.50% BSG. t1, t7, and t20 refer to 1, 7, and 20 days after production, respectively.

The results of the texture analysis are reported in Figure 1. The H bars (Figure 1a,b) showed a significantly softer and more deformable texture than the C bars (Figure 1c,d). The H bars contained higher levels of brewer’s spent grain (BSG) in their formulation, which may have led to bars with uneven distribution, resulting in empty spaces and contributing to a softer texture [22]. Moreover, for the C bars, the higher binder/cereal ratio, together with the binder formulation rich in sugar and starch, known for their thickening effect [18], could have created a more rigid structure in these samples.

Focusing on the H bars at t1, both fracturability (Figure 1a) and deformability (Figure 1b) were slightly influenced by the BSG amount and showed average values of 6.1 N and 6.9 mm, respectively. Only H3 showed significantly lower values of fracturability in comparison to the other samples (Figure 1a). Indeed, the higher BSG amount of the H3 sample might have weakened the bar structure, resulting in a decrease in both texture parameters [22]. A different trend can be observed at t7 and t20: the deformability values of the different H bars showed increased values as BSG content increased. This result might be associated with the presence of hydrated fibrous material that enhances the bars’ flexibility [53].

On the contrary, for C samples, fracturability values measured at t1 increased when increasing the amount of BSG in the formulation (Figure 1c), ranging from ~28 N to ~96 N for CSTD and C3 samples, respectively. This trend was opposite compared to the one observed for MC and *a*_w_ for C samples (Table 6), which highlights the central role of ingredient hydration on the texture of this kind of product. According to Ergun et al. (2010) [54], a product’s water content has a significant impact on its texture: formulations with lower moisture content result in harder products. The deformability values at t1 showed no significant variation among samples with different BSG levels for both H bars and C bars, showing average values of 6.93 mm and 3.69 mm, respectively.

During storage, fracturability values of HSTD and CSTD showed increasing trends. This hardening phenomenon could be attributed to reduced MC and *a*_w_ values, as noted by Su-Ah et al. (2018) [49]. The authors highlighted that processes like water migration, sugar and starch crystallization, and fat hydrolysis could potentially impact the textural properties of cereal bars during storage. For BSG cereal bars, fracturability changed during storage as a function of the BSG addition level and the type of binding system. Considering H bars, all the samples exhibited an increase in fracturability from t1 to t7, while at t20, fracturability values decreased in H1 and H2 and remained constant in H3. It may be hypothesized that, during the first days of storage, cereal bars showed a surface water loss which resulted in the hardening of the outer layers (Table 6). However, after 7 days, the internal water of cereal bars tends to migrate towards the outer layers and is absorbed by the cereals, which makes the texture of the bar softer [53]. H3, which had a much higher concentration of BSG than the other two samples (H1 and H2), probably bound water more strongly, thus preventing softening over time. With regard to C samples, C1 behaved similarly to H1, while C2 and C3 did not show any fracturability change during storage. Regarding deformability, HSTD, H1, and H2 showed a significant reduction at t7. The extent of this decrease in deformability changed as a function of the BSG content. The parameter remained constant at t20. The increased rigidity of the structure over time could be associated with the binder hardening [49]. Differently, H3 showed an increase in deformability at t7 followed by a decrease at t20. This result might be associated with the presence of fibrous material in BSG samples, which enhances the bars’ flexibility. The C samples were more stable in terms of deformability, showing no significant differences during storage, except for C3, which revealed a significant decrease in this parameter at t20.

### 3.4. Color

**Figure 2:**
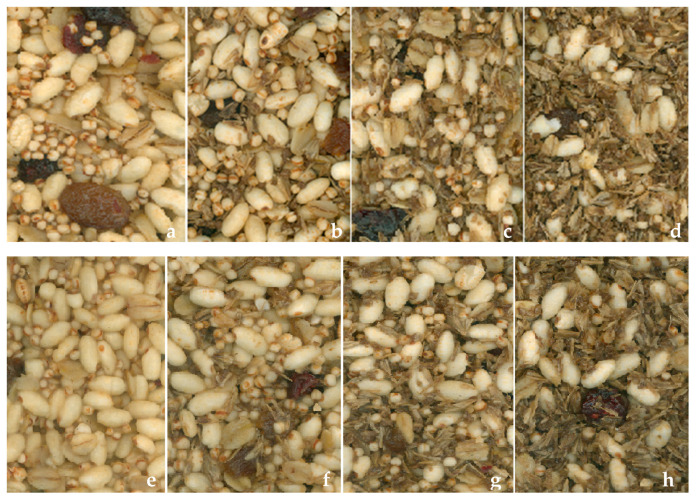
Digital images of H and C cereal bars stored one day after the production process. Note: (**a**) HSTD = standard cereal bar with H binding system and without BSG; (**b**) H1= cereal bar with H binding system and 8.46% BSG; (**c**) H2 = cereal bar with H binding system and 12.69% BSG; (**d**) H3 = cereal bar with H binding system and 21.15% BSG; (**e**) CSTD = standard cereal bar with C binding system and without BSG; (**f**) C1 = cereal bar with C binding system and 3.87% BSG; (**g**) C2 = cereal bar with C binding system and 7.74% BSG; (**h**) C3 = cereal bar with C binding system and 15.50% BSG.

The color parameters obtained from the digital images (Figure 2) of the cereal bars during storage are reported in Table 7.

Increasing the amount of BSG in the formulation led to a general darkening of the color, which was outlined by the reduction in the color parameters L*, a*, and b*. This phenomenon can be observed at t1 for both H and C samples. On the other hand, the storage time had a smaller influence compared to the effect of the BSG incorporation on the cereal bars’ color. This is also highlighted by the ΔE results, which showed higher values when the BSG-containing samples were compared to the STD samples, rather than the values obtained from the comparison performed as a function of storage time in each cereal bar formulation (Table 7). Musatto et al. (2006) reported that BSG was brownish in color when moist and thus, they suggested using it in off-white products [7]. Moreover, the darker color of BSG is strongly influenced by the type of malt used and the brewing treatment. It is reported that high malt kilning temperatures (80–250 °C) may induce Maillard reactions, caramelization, and pigment degradation, thus reducing BSG brightness [50]. The slight color modifications observed on the cereal bars during storage may be attributable to the water evaporation phenomenon, which led to the crystallization of sugar and fats present in the binders, and/or to the starch retrogradation phenomenon, which altered the light angle diffraction. The correlation between MC and color parameters in the two cereal bar samples, at each storage time and BSG level, was tested. In H bars, a positive correlation was found between MC and the color parameters L* (*p* < 0.05; R = 0.631), a* (*p* < 0.01; r = 0.856), and b* (*p* < 0.05; r = 0.646); however, only b* was positively correlated (*p* < 0.05; r = 0.612) with MC for C samples.

### 3.5. Liking and Purchasing Intent

Sensory analysis was performed on two selected cereal bars, i.e., H2-t20 and C2-t20 samples (as described in Section 2.3). Table 8 displays the average liking scores as a function of product type (H2-t20 and C2-t20) and consumer group (blind and informed group). The results showed that the information on the use of BSG did not influence consumers’ liking either in the expected or actual conditions. In addition, no differences were highlighted between the two cereal bars (i.e., H2-t20 and C2-t20 samples). However, it should be noted that average scores for expected liking are positive for both products and in both groups; thus, regardless of the information provision, consumers expected a positive sensory experience. After tasting, the liking scores significantly increased for both products and in both groups, which highlights the central role of the sensory experience over information provision.

Similarly, the purchase intent scores (5-pt scale) did not differ between products, either when tested in the blind (H bar = 2.98 ± 1.1, C-bar= 3.00 ± 1.07) or in the informed (H bar = 3.06 ± 0.97, C-bar= 2.94 ± 1.24) condition.

On the other hand, when investigating participants’ purchasing intention for a general energy bar made with by-products of the beer or cereal industry, *t*-tests showed significant differences between groups, with the informed group having a higher purchasing intention for both the cereal by-product bar (blind = 3.30 ± 0.86 and informed = 3.83 ± 0.71; (t(72) = −2.8241, *p* = 0.006)) and the beer by-product bar (blind = 3.32 ± 0.91 and informed= 3.80 ± 0.90; (t(72) = −2.3224, *p*= 0.023)).

Despite past research [23] showing that the intention to purchase upcycled food is stronger when the product and its benefits are communicated more concretely than abstractly, our results did not show significant differences between the blind and the informed groups. In our study, for purchasing intention, as for liking scores, the sensory experience revealed a higher effect than the information provision since participants’ willingness to purchase the proposed cereal bars did not differ between groups. Nevertheless, when investigating the purchasing intents of a hypothetical energy bar made with by-products (thus, without tasting the actual product) the information on upcycled ingredients increased consumers’ purchasing intention. This result shows that consumers are still largely unaware of what upcycled food is and, thus, strategies to effectively inform consumers about the benefits of using such ingredients are critical to ensure market acceptance [23].

Our results highlighted the need to make upcycled snacks more appealing in terms of sensory experience.

### 3.6. Sensory Attributes

Paired *t*-tests were used to explore sensory characteristics (i.e., aspect, color, texture, crunchiness, chewiness, adhesiveness, taste, sweetness, and smell) of the two cereal bars (i.e., H2-t20 and C2-t20 bars) measured on a 9-point hedonic scale (1 = dislike extremely, 9 = like extremely) (Table 9).

Aspect, color, and smell were the sensory attributes that received the lowest scores for both H2-t20 and C2-t20 bars in both blind and informed conditions. The negative impact of adding BSG to food formulations on these attributes has already been reported by other authors. For example, Stelick et al. (2021) [22] found that the presence of visible grain fibers/bran on BSG cereal bars’ surface negatively affected their perceived appearance. This result is in line with our study (see Figure 2). Moreover, the same authors reported that the greenish/gray hue conferred by BSGs affected the color liking; indeed, the penalty analysis highlighted color as the only penalizing attribute for the tested BSG cereal bars’ formulations [22]. Cheng et al. (2024) reported that adding BSG above 6% in bread formulations reduced the sensory scores for color, texture, and flavor due to the darkening, hardening, and development of altered odor notes [55]. On the other hand, Farcas et al. (2021) [50] explored the impact of incorporating five different types of BSG at a 20% ratio to wheat flour in cookie production. The inclusion of BSG, particularly the light and dark malt blends, enhanced the sensory qualities of the cookies. This result was attributed to their rich color, improved texture, and distinctive aroma profile. Chemical analyses of volatile compounds revealed that the BSG samples were characterized by higher levels of aldehyde compounds compared to the control sample formulated without the addition of BSG. The higher levels of aldehyde compounds contributed to notes of malt, fruitiness, dried fruits, nuts, chocolate, cocoa, fat, and caramel, which created a pleasant consumer odor perception [50].

Comparing the H2-t20 and C2-t20 bars, aspect and color were the only two sensory attributes for which H2-t20 bars received a significantly lower score in comparison to C2-t20 bars when tested in blind conditions. Table 7 showed that the color of the tasted H2-t20 bars was in general lighter than that of the C2-t20 bars, with higher values for all three colorimetric parameters. Similar results were observed by Aigster et al. (2011), who detected lower color sensory scores for granola bars incorporating resistant starch, resulting in a lighter color in comparison to the control [56]. The higher percentage of BSG contained in H bars in comparison to the C bars (Table 1) may have affected the appearance perception, as already stated by Stelick et al. (2021) [22]. Providing information about the samples flattened out the observed differences.

Overall, no significant differences were identified when comparing the sensory scores of cereal bars tested under blind and informed conditions. Crofton and Scannell (2020) [57] conducted detailed interviews to investigate consumer preferences regarding four snack product concepts made with BSG. Their research revealed that consumers did not perceive the incorporation of BSG in snack foods as the only way to achieve the desired outcome. The authors concluded that manufacturers play a crucial role not only in developing sustainable snacks but also in ensuring that important attributes like taste, convenience, or price are not compromised.

### 3.7. CATA Test

The results from the CATA test (Table 10) indicated that the two main attributes selected for H2-t20 bars were ‘natural’ and ‘healthy’, while ‘crunchy’ and ‘sweet’ were the most frequently chosen attributes for the C2-t20 bars.

Only under the blind condition, significant differences for the attributes ‘natural’ and ‘healthy’ were observed among H and C bars. On the other hand, significant differences for the attribute ‘crunchy’ were observed between the two samples only under the informed condition, whereas for the ‘sweet’ attribute, significant differences were detected under both blind and informed conditions. Stelick et al. (2021) found that, when applying a CATA test, cereal bars containing around 12% BSG were most frequently described as ‘natural’ and ‘healthy’ compared to a commercial control sample without BSG [22]. Curutchet et al. (2022) investigated consumer responses to cakes enriched with apple pomace as a sustainable source of fiber [24]. These authors applied the CATA test and found that the fiber-enriched cake showed a higher frequency for the terms ‘homemade’ and ‘healthy’, which were also considered among the most important drivers of liking. In our study, the lower BSG content of the C2-t20 bars (7.74%) in comparison to the H2-t20 bars (12.69%) (Table 1) may explain the reason why only these latter were perceived as ‘natural’ and ‘healthy’. The C2-t20 samples were most commonly described by panelists as having a ‘crunchy’ and ‘sweet’ sensory profile. From the texture analysis, the C bars were harder than the honey ones (Figure 1). This instrumental result may be associated with the perceived crunchiness, while the high sugar content and the presence of white chocolate in the binder (Table 1) may explain the perceived sweetness.

## 4. Conclusions and Future Research

Due to recent technological advancements in processing techniques, there is emerging research in the context of the circular economy to reuse by-products of food processing as ingredients, including the use of BSG.

This study demonstrates that BSG can be successfully incorporated into cereal bar formulations as a valuable source of fiber, proteins, and minerals. In addition, the inclusion of certain levels of BSG allowed the cereal bars to meet the nutritional claim “with high fiber content”, according to European legislation. However, increasing the amount of BSG resulted in a general darkening of the color and negatively impacted the texture aspect and smell, particularly for the H bars, which contained a higher BSG content and lower solid ingredient/binder ratio than the C bars. From the CATA tests, H bars were perceived as healthy and natural—presumably given the higher BSG content—whereas C bars as crunchy and sweet—possibly because of the binder type.

The sensory experience of cereal bars significantly outperformed the information provided about the addition of BSG in the products. Participants’ expected liking was lower than their actual liking after tasting; moreover, liking and willingness to purchase the cereal bars did not differ between the blind and informed groups. On the other hand, the information increased the purchasing intention for a hypothetical energy bar made with by-products.

This study has a few limitations that could be addressed in future research. First, we considered a short-term storage period of cereal bars (20 days); thus, further research should investigate the long-term storage stability and shelf life of BSG-enriched cereal bars to assess their commercial viability. In addition, the sample size for the consumer study was relatively small (n = 76). Conducting a larger-scale consumer study with a more diverse population could provide more robust insights into consumer acceptance and purchasing behavior.

These limitations highlight the need for further research to fully understand the potential of BSG in cereal bar formulations and to address the concerns related to sensory attributes and long-term storage stability.

## Figures and Tables

**Table 1:** Ingredients used to produce the binding systems.

Honey Binding System	Ingredients	For 100 g Cereal Bar	For Each Cereal Bar (29.55 g)
H binder	Margarine (Conad)	25.38 g	7.50 g
	Cane sugar (Eridania)	12.69 g	3.75 g
	Honey (Ambrosoli)	6.77 g	2.00 g
	Soy lecithin (Vital Nature)	0.17 g	0.05 g
	Water (T = 25 °C, Conad)	4.23 g	1.25 mL
**Chocolate** **Binding system**	**Ingredients**	**For 100 g cereal bar**	**For each cereal bar (32.30 g)**
C binder	Margarine (Conad)	23.22 g	7.50 g
	Cane sugar (Eridania)	13.93 g	4.50 g
	Sunflower oil (Conad)	13.93 g	4.50 g
	White chocolate (Conad) *	6.20 g	2.00 g
	Corn starch (Unilever)	2.32 g	0.75 g
	Soy lecithin (Vital Nature)	0.15 g	0.05 g
	Water (T = 25 °C, Conad)	9.30 mL	3.00 mL

* White chocolate formulation: sugar, cocoa butter, whole milk powder, emulsifiers, soy lecithin, natural aroma.

**Table 2:** Solid ingredients used for the two cereal bar formulations, namely H and C bars.

Cereal Bar	BSG (g)	Puffed Rice (g)	Puffed Quinoa (g)	Oat Flakes (g)	Dried Fruits (g)	Total Solid Ingredients (g)
**HSTD**	0	3.50	1.25	4.00	6.25	15.00
**H1**	2.5 (8.46%)	3.00	1.00	3.25	5.25	15.00
**H2**	3.75 (12.69%)	2.75	0.75	3.00	4.75	15.00
**H3**	6.25 (21.15%)	2.00	0.50	2.50	3.75	15.00
**CSTD**	0	4.00	1.00	2.50	2.50	10.00
**C1**	1.25 (3.87%)	3.50	1.25	2.00	2.00	10.00
**C2**	2.50 (7.74%)	3.00	1.00	1.75	1.75	10.00
**C3**	5.00 (15.48%)	2.00	0.50	1.25	1.25	10.00

Note: HSTD = standard cereal bar with H binding system and without BSG; H1 = cereal bar with H binding system and 8.46% BSG; H2 = cereal bar with H binding system and 12.69% BSG; H3 = cereal bar with H binding system and 21.15% BSG; CSTD = standard cereal bar with C binding system and without BSG; C1 = cereal bar with C binding system and 3.87% BSG; C2 = cereal bar with C binding system and 7.74% BSG; C3 = cereal bar with C binding system and 15.50% BSG.

**Table 3:** Proximate composition of brewer’s spent grain (BSG).

Macronutrients	g/100 g dm (%)
Fiber	58.33
Protein	20.89
Lipid	13.11
Saturated lipid	3.40
Unsaturated lipid	9.23
Carbohydrates	4.80
Of which sugars	1.94
Ash	4.27
**Minerals**	**mg/kg dm**
Na	26.00
Tot. P	5638.00
Ca	4307.00
Tot. S	2398.00
Mg	2086.00
K	343.00
Fe	133.40
Mn	40.20
Zn	86.50
Al	136.15
Cu	8.40
Co	0.014
**Vitamins**	**mg/kg dm**
Thiamine (B1 vitamin)	2.52
Riboflavin (B2 vitamin)	0.29
Cobalamin (B12 vitamin)	0.05
Pantothenic acid (B5 vitamin)	0.18
Pyridoxine (B6 vitamin)	1.21
PP vitamin (B3 vitamin)	
Nicotinic acid	38.44
Nicotinamide	6.03

Note: Data are expressed as percentages of dry matter (dm). The values of relative standard deviation were below 7%.

**Table 4:** Amino acid profile of BSG sample.

Amino Acid (AA)	mg/100 g of BSG	mg/100 g Protein
Glucosammic acid	0.000	0.00
Aspartic acid	0.336	7.91
Serine + asparagine	0.203	4.78
Glutammic acid	0.860	20.24
Glycine	0.174	4.09
Istidine + glutamine	0.091	2.14
Arginine	0.200	4.71
Threonine	0.148	3.48
β-alanine	0.004	0.09
Alanine	0.211	4.96
Methionine sulfone	0.000	0.00
Proline	0.357	8.40
g-aminobutirric	0.020	0.47
Proline Bi	0.000	0.00
α-aminobutirric	0.000	0.00
Cysteine	0.125	2.94
Tyrosine	0.050	1.18
Valine	0.229	5.39
Methionine	0.099	2.33
Ornitine	0.003	0.07
Lysine	0.195	4.59
Iso-leucine	0.141	3.32
Leucine	0.321	7.55
Phenilalanine	0.226	5.32
Tryptophan	0.002	0.05
Total	3.995	93.95

Note: The values of relative standard deviation were below 5%.

**Table 5:** Amount of fiber provided by each ingredient for the H and C cereal bar formulations.

Cereal Bars	Ingredient’s Fiber Content per 100 g Cereal Bar (g)					Grams of Fiber in 100 g Cereal Bar	Grams of Fiber per Cereal Bar	Nutrition Claim
	BSG	Puffed Rice	Puffed Quinoa	Oat Flakes	Dried Fruits			
HSTD	0.00	0.39	0.15	1.49	1.18	3.21	0.95	Fiber source
H1	4.93	0.34	0.12	1.21	0.99	7.59	2.24	High fiber
H2	7.40	0.31	0.09	1.12	0.90	9.82	2.90	High fiber
H3	12.34	0.22	0.06	0.93	0.71	14.26	4.21	High fiber
CSTD	0.00	0.41	0.11	0.85	0.43	1.80	0.58	No claim
C1	2.26	0.36	0.14	0.68	0.35	3.78	1.22	Fiber source
C2	4.51	0.31	0.11	0.60	0.30	5.83	1.88	High fiber
C3	9.03	0.20	0.05	0.43	0.22	9.93	3.21	High fiber

Note: HSTD = standard cereal bar with H binding system and without BSG; H1 = cereal bar with H binding system and 8.46% BSG; H2 = cereal bar with H binding system and 12.69% BSG; H3 = cereal bar with H binding system and 21.15% BSG; CSTD = standard cereal bar with C binding system and without BSG; C1 = cereal bar with C binding system and 3.87% BSG; C2 = cereal bar with C binding system and 7.74% BSG; C3 = cereal bar with C binding system and 15.50% BSG.

**Table 6:** Moisture content (MC) and water activity (*a*_w_) of binders and cereal bars.

	MC (%)					*a*_w_				
Binders	H	70.73 ± 0.65		C	72.75 ± 1.10	H	0.35 ± 0.01		C	0.68 ± 0.01
Cereal Bars	t1		t7		t20	t1		t7		t20
HSTD	9.99 ± 1.41 ^aA^		9.36 ± 1.05 ^aA^		7.77 ± 1.56 ^aA^	0.45 ± 0.03 ^aA^		0.40 ± 0.02 ^aB^		0.26 ± 0.00 ^bC^
H1	9.26 ± 0.92 ^aA^		6.60 ± 1.34 ^abB^		6.29 ± 0.57 ^aB^	0.44 ± 0.03 ^abA^		0.28 ± 0.01 ^bC^		0.36 ± 0.02 ^aB^
H2	8.22 ± 0.95 ^aA^		6.92 ± 0.87 ^abA^		6.56 ± 1.05 ^aA^	0.39 ± 0.01 ^bcA^		0.29 ± 0.01 ^bB^		0.37 ± 0.01 ^aA^
H3	8.07 ± 0.98 ^aA^		6.17 ± 0.94 ^bA^		6.05 ± 1.29 ^aA^	0.37 ± 0.00 ^cA^		0.29 ± 0.01 ^bC^		0.36 ± 0.01 ^aB^
CSTD	10.91 ± 0.96 ^aA^		7.63 ± 2.37 ^aAB^		6.01 ± 0.58 ^aB^	0.73 ± 0.02 ^aA^		0.62 ± 0.04 ^aB^		0.29 ± 0.01 ^bC^
C1	9.06 ± 0.22 ^bA^		4.84 ± 0.28 ^abB^		4.17 ± 1.16 ^abB^	0.60 ± 0.06 ^abA^		0.51 ± 0.08 ^aA^		0.29 ± 0.01 ^bB^
C2	8.31 ± 0.75 ^bA^		4.97 ± 1.14 ^abB^		4.65 ± 1.45 ^abB^	0.59 ± 0.08 ^bcA^		0.25 ± 0.02 ^bB^		0.31 ± 0.01 ^aB^
C3	6.67 ± 0.17 ^cA^		3.90 ± 0.54 ^bB^		3.02 ± 0.92 ^bB^	0.45 ± 0.04 ^cA^		0.23 ± 0.02 ^bC^		0.30 ± 0.01 ^abB^

Note: Data expressed as mean values ± standard deviations. Different small letters in the same column indicate significant differences (*p* < 0.05) among formulations at the same storage time. Different capital letters in the same row indicate significant differences (*p* < 0.05) for the same formulation at different storage times. HSTD = standard cereal bar with H binding system and without BSG; H1 = cereal bar with H binding system and 8.46% BSG; H2 = cereal bar with H binding system and 12.69% BSG; H3 = cereal bar with H binding system and 21.15% BSG; CSTD = standard cereal bar with C binding system and without BSG; C1 = cereal bar with C binding system and 3.87% BSG; C2 = cereal bar with C binding system and 7.74% BSG; C3 = cereal bar with C binding system and 15.50% BSG. t1, t7, and t20 refer to 1, 7, and 20 days after production, respectively.

**Table 7:** Color parameters of cereal bars during storage.

	L*	a*	b*	ΔE_time_	ΔE_formulation_
HSTD-t1	64.09 ± 3.34 ^aA^	2.36 ± 0.82 ^aA^	28.49 ± 1.36 ^aA^	-	-
H1-t1	51.67 ± 3.36 ^bB^	1.51 ± 0.24 ^bA^	23.75 ± 0.81 ^bB^	-	13.35 ± 3.37
H2-t1	53.27 ± 3.48 ^bA^	1.57 ± 0.36 ^abA^	24.10 ± 0.92 ^bA^	-	11.74 ± 3.53
H3-t1	49.13 ± 1.51 ^bA^	1.60 ± 0.37 ^abA^	22.90 ± 0.36 ^bA^	-	16.00 ± 1.51
HSTD-t7	65.19 ± 2.64 ^aA^	1.98 ± 0.44 ^aA^	28.76 ± 0.89 ^aA^	2.40 ± 1.65	-
H1-t7	54.48 ± 1.08 ^bAB^	1.28 ± 0.63 ^abA^	25.17 ± 0.57 ^bA^	3.30 ± 0.90	11.35 ± 0.92
H2-t7	53.23 ± 2.99 ^bA^	1.42 ± 0.62 ^abA^	24.16 ± 0.72 ^bA^	2.35 ± 1.80	12.85 ± 3.02
H3-t7	43.93 ± 1.88 ^cB^	0.98 ± 0.37 ^bB^	22.05 ± 0.85 ^cAB^	5.34 ± 1.99	22.32 ± 2.05
HSTD-t20	61.07 ± 2.51 ^aA^	1.58 ± 0.63 ^aA^	26.84 ± 0.94 ^aB^	4.01 ± 1.83	-
H1-t20	56.76 ± 2.34 ^abA^	1.47 ± 0.27 ^aA^	25.13 ± 0.78 ^bA^	5.33 ± 2.34	4.79 ± 2.09
H2-t20	53.19 ± 1.66 ^bA^	1.09 ± 0.09 ^abA^	23.96 ± 0.54 ^bA^	1.54 ± 0.48	8.42 ± 1.65
H3-t20	44.09 ± 2.89 ^cB^	0.60 ± 0.26 ^bB^	21.54 ± 0.43 ^cB^	5.38 ± 2.80	17.83 ± 2.85
	L*	a*	b*	**ΔE_time_**	**ΔE_formulation_**
CSTD-t1	62.55 ± 1.61 ^aA^	1.62 ± 0.34 ^aA^	28.22 ± 0.43 ^aA^	-	-
C1-t1	56.85 ± 2.23 ^bA^	0.89 ± 0.14 ^bA^	24.56 ± 0.84 ^bA^	-	6.87 ± 2.20
C2-t1	55.11 ± 2.47 ^bA^	0.57 ± 0.19 ^bcA^	23.21 ± 0.60 ^cA^	-	9.10 ± 2.26
C3-t1	47.56 ± 2.37 ^cA^	0.31 ± 0.26 ^cA^	21.19 ± 0.60 ^dA^	-	16.62 ± 2.34
CSTD-t7	64.57 ± 1.56 ^aA^	1.63 ± 0.45 ^aA^	28.49 ± 0.81 ^aA^	2.22 ± 1.53	-
C1-t7	58.22 ± 1.83 ^bA^	0.64 ± 0.36 ^abA^	24.43 ± 0.82 ^bA^	2.17 ± 0.91	7.65 ± 1.80
C2-t7	53.93 ± 3.52 ^bA^	0.05 ± 0.34 ^abA^	22.04 ± 0.57 ^bAB^	2.35 ± 1.95	12.67 ± 3.01
C3-t7	46.48 ± 2.65 ^cA^	0.37 ± 0.25 ^bA^	21.07 ± 0.91 ^cA^	2.37 ± 1.61	19.60 ± 2.74
CSTD-t20	65.19 ± 2.44 ^aA^	1.71 ± 0.46 ^aA^	28.22 ± 0.72 ^aA^	3.00 ± 2.07	-
C1-t20	55.53 ± 1.58 ^bA^	0.93 ± 0.30 ^bA^	24.08 ± 0.50 ^bA^	1.66 ± 1.38	10.55 ± 1.57
C2-t20	52.35 ± 3.44 ^bA^	0.18 ± 0.58 ^cA^	21.80 ± 1.13 ^cB^	4.16 ± 2.09	14.47 ± 3.54
C3-t20	47.49 ± 2.09 ^cA^	0.39 ± 0.14 ^bcA^	20.60 ± 0.47 ^cA^	1.79 ± 1.09	19.33 ± 2.03

Note: Data expressed as mean values ± standard deviations. Different small letters in the same column indicate significant differences (*p* < 0.05) among formulations at the same storage time. Different capital letters in the same column indicate significant differences (*p* < 0.05) for the same formulation at different storage times. HSTD = standard cereal bar with H binding system and without BSG; H1 = cereal bar with H binding system and 8.46% BSG; H2 = cereal bar with H binding system and 12.69% BSG; H3 = cereal bar with H binding system and 21.15% BSG; CSTD = standard cereal bar with C binding system and without BSG; C1 = cereal bar with C binding system and 3.87% BSG; C2 = cereal bar with C binding system and 7.74% BSG; C3 = cereal bar with C binding system and 15.50% BSG. t1, t7, and t20 refer to 1, 7, and 20 days after production, respectively.

**Table 8:** Expected and actual liking of H2-t20 and C2-t20 cereal bars under blind and informed conditions.

Variable	Blind		Informed	
	H2-t20	C2-t20	H2-t20	C2-t20
Expected liking	4.07 ± 1.17 ^aB^	4.29 ± 1.19 ^aB^	4.49 ± 0.98 ^aB^	4.54 ± 1.07 ^aB^
Actual liking	5.05 ± 1.41 ^aA^	5.37 ± 1.04 ^aA^	5.20 ± 1.12 ^aA^	5.23 ± 1.44 ^aA^

Note: Data expressed as mean values ± standard deviations, calculated on a 7-point hedonic scale (from 1 = dislike extremely to 7 = like extremely). Different lowercase letters, within a row, indicate significant differences (*p* < 0.05—ANOVA) between the two groups (blind and informed) and between formulations. Different capital letters, within a column, indicate significant differences (*p* < 0.05—paired sample *t*-test) between the two conditions (expected vs. actual) within the same product sample and within groups (blind and informed). H2 = cereal bar with H binding system and 12.69% BSG; C2 = cereal bar with C binding system and 7.74% BSG; C3 = cereal bar with C binding system and 15.50% BSG. t20 refers to 20 days after production.

**Table 9:** Sensory rating of the H2-t20 and C2-t20 cereal bars under blind and informed conditions.

Product/ Sensory Attributes	Blind		Informed	
	H2-t20	C2-t20	H2-t20	C2-t20
Aspect	4.73 ± 1.57 ^bA^	5.22 ± 1.64 ^aA^	5.20 ± 1.89 ^aA^	5.26 ± 1.74 ^aA^
Color	4.73 ± 1.55 ^bA^	5.22 ± 1.67 ^aA^	5.14 ± 1.57 ^aA^	5.06 ± 1.51 ^aA^
Texture	6.10 ± 1.66 ^aA^	6.34 ± 1.24 ^aA^	6.03 ± 1.40 ^aA^	6.09 ± 1.52 ^aA^
Crunchiness	6.34 ± 1.74 ^aA^	6.81 ± 1.25 ^aA^	5.94 ± 1.70 ^aA^	6.49 ± 1.70 ^aA^
Chewiness	5.68 ± 1.76 ^aA^	6.20 ± 1.32 ^aA^	6.00 ± 1.83 ^aA^	6.09 ± 1.58 ^aA^
Adhesiveness	5.42 ± 1.55 ^aA^	5.63 ± 1.24 ^aA^	5.89 ± 1.41 ^aA^	5.77 ± 1.46 ^aA^
Taste	6.49 ± 1.86 ^aA^	6.54 ± 1.42 ^aA^	6.80 ± 1.17 ^aA^	6.37 ± 1.95 ^aA^
Sweetness	6.51 ± 1.49 ^aA^	6.22 ± 1.48 ^aA^	6.77 ± 1.53 ^aA^	6.03 ± 1.72 ^aA^
Smell	4.83 ± 1.83 ^aA^	5.22 ± 1.57 ^aA^	4.97 ± 1.90 ^aA^	5.00 ± 1.72 ^aA^

Note: Data expressed as mean values ± standard deviations, calculated on a 9-point hedonic scale (from 1 = dislike extremely to 9 = like extremely). Different lowercase letters within a row denote product sensory attributes with significant differences between the cereal bar types (H2-t20 and C2-t20) according to paired *t*-tests (*p* < 0.05); Different uppercase letters within a row denote product sensory attributes with significant differences between groups (blind and informed), according to unpaired *t*-tests (*p* < 0.05). H2 = cereal bar with H binding system and 12.69% BSG; C2 = cereal bar with C binding system and 7.74% BSG; C3 = cereal bar with C binding system and 15.50% BSG. t20 refers to 20 days after production.

**Table 10:** Check-all-that-apply results of the H2-t20 and C2-t20 cereal bars under blind and informed conditions.

Product Descriptors	Blind		Informed	
	H2-t20	C2-t20	H2-t20	C2-t20
Crunchy	0. 561 ^a^	0. 732 ^a^	0.457 ^b^	0.743 ^a^
Sweet	0. 488 ^b^	0.805 ^a^	0.429 ^b^	0.743 ^a^
Natural	0.683 ^a^	0.488 ^b^	0.543 ^a^	0.429 ^a^
Tasty	0.366 ^a^	0.317 ^a^	0.371 ^a^	0.314 ^a^
Healthy	0.659 ^a^	0.488 ^b^	0.543 ^a^	0.514 ^a^
Gummy	0.146 ^a^	0.146 ^a^	0.200 ^a^	0.086 ^a^
Soft	0.195 ^a^	0.146 ^a^	0.200 ^a^	0 ^b^
Sticky	0.268 ^a^	0.268 ^a^	0.143 ^a^	0.143 ^a^
Artificial	0.024 ^a^	0.098 ^a^	0 ^b^	0.086 ^a^
Colored	0.024 ^a^	0.024 ^a^	0.114 ^a^	0.029 ^a^
Intriguing	0.171 ^a^	0.171 ^a^	0.143 ^a^	0.114 ^a^
Practical	0.220 ^a^	0.317 ^a^	0.229 ^a^	0.200 ^a^
Unpleasant	0.122 ^a^	0.049 ^a^	0.086 ^a^	0.143 ^a^
Bland	0.073 ^a^	0.073 ^a^	0.057 ^a^	0 ^a^

Note: Data expressed as percentages calculated on frequency counts. Different superscripts indicate significant differences in the selected sensory attributes (*p* < 0.05) between the two cereal bars and within the group (blind and informed). H2 = cereal bar with H binding system and 12.69% BSG; C2 = cereal bar with C binding system and 7.74% BSG; C3 = cereal bar with C binding system and 15.50% BSG. t20 refers to 20 days after production.

## Data Availability

The original contributions presented in the study are included in the article, further inquiries can be directed to the corresponding author.
